# Choking or Delivering Under Pressure? The Case of Elimination Games in NBA Playoffs

**DOI:** 10.3389/fpsyg.2018.00979

**Published:** 2018-06-12

**Authors:** Elia Morgulev, Yair Galily

**Affiliations:** Sport, Media and Society (SMS) Research Lab, Sammy Ofer School of Communications Interdisciplinary Center (IDC) Herzliya, Herzliya, Israel

**Keywords:** expected utility theory, choking, stress, pressure, NBA, playoffs, basketball

## Abstract

Neoclassical economic theories foretell that individuals exert the most effort, and consequently produce their best performances, when the net returns to effort are highest. We scanned through 33 NBA seasons and analyzed 1930 playoffs games in order to test this prediction. Analysis of win probabilities in games where one of the two teams faces elimination from the playoffs, demonstrated that the threat of severe losses didn’t lead to elevated level of performance. While previous studies analyzed mainly single-level performance in a stable environment, our results shed light on collective performance in a dynamic setting. These findings can be applicable to other realms as we suggest that managers should refrain from deliberate building of high-pressure environments with hopes of achieving performance enhancement effect among their groups.

## Introduction

In 210 BC, a prominent Chinese warlord named Xiang Yu led his rebel forces across the Yangtze River to battle against the Qin dynasty. Camping by the water for the night, his troops awakened in the dawn to find that their ships were burning. Firstly, they hurried to their feet to fight off their attackers, only to discover that it was Xiang Yu himself who had set their ships on fire.

Ariely (2009, p. 183) described this ancient tactical maneuver where troops without ships for the retreat were left with only one choice – to fight desperately and move forward, or perish. From the perspective of “expected utility” theory Xiang Yu simply shifted the payoff matrix and created a sharp asymmetry between the costs of loss for soldiers of the confronting armies; asymmetry that probably had a tremendous focusing effect on Xiang’s forces, which mobilized all their mental and physical resources and thereon crushed their enemy in nine consecutive battles.

Expected utility theory already has been applied for analysis of behavior in military conflict situations (De Mesquita, 1980). For instance, an example of expected utility reasoning is found in Maier’s (1988) analysis of conflicts that led to World War I.

As for behavior under conflict, sports provide us with a setting where high-profile agents invest their talent, effort, and expertise to outperform their opponents in situations with extremely high stakes and measurable outcomes. Consequently, sports became a fertile ground for academic inquiry. This may be evident in a stream of economic and psychological research that use sports as a lab to study human behavior (e.g., Gilovich et al., 1985; Duggan and Levitt, 2002; Palacios-Huerta, 2003; Dohmen, 2008; Morgulev et al., 2014).

In line with this stream, Elaad et al. (2015) used the setting of sports to investigate corruption, alongside some meaningful findings about corruption elicited in this research, these researchers also pointed out that football teams that struggle to avoid relegation to a lower division probably exert a higher effort than the opponent team for which the match is less critical. According to the expected utility theory, the cost of loss is much larger for the former team. Elaad et al.’s (2015) premise corresponds with previous theoretical literature that suggests that competitors are expected to increase their effort in critical matches (Szymanski, 2003; Scarf and Shi, 2008).

Another relevant phenomenon was reported in a study on golf (Pope and Schweitzer, 2011), where every hole has a number of strokes associated with it, and the par number provides a reference point for a satisfying performance. For a professional golfer, a birdie (one stroke under par) is a gain, and a bogey (one stroke over par) is a loss. The researchers compared the situation where the player is putting to avoid a bogey, with a more favorable setting where the player is aiming to achieve a birdie. A hypothesis derived from the concept of loss aversion suggested that players would try harder when putting for par (to avoid a bogey) than when putting for a birdie. An analysis of more than 2.5 million putts supported that prediction.

Nobel Laureate Daniel Kahneman referred to this finding in his 2011 book: “These fierce competitors certainly do not make a conscious decision to slack off on birdie putts, but their intense aversion to a bogey apparently contributes to extra concentration on the task at hand” (Kahneman, 2011, p. 304).

However, one should not overlook the considerable mass of literature that suggests that stressful environment may not boost but rather hinder performance. For instance, Dohmen (2008) pointed out that “high rewards or the threat of severe punishment might sometimes be perceived as pressuring and lead to poor performance” (p. 636). This phenomenon is known as choking under pressure, and it was documented both in professional football and basketball (Dohmen, 2008; Apesteguia and Palacios-Huerta, 2010; Cao et al., 2011).

Social scientists have stated that pressure can motivate, but it can also generate too much self-focus (thinking about the details of how one should accomplish a goal, as opposed to ‘just doing it’). Goldman and Rao (2012) have implicated self-focus to analyze pressure-associated performance declines in basketball.

While experiments allow one to control for confounding factors and isolate the choking phenomenon, the results may not always be applicable to real world situations. As we asserted earlier and as Hickman and Metz (2015) emphasize, sports are often studied as they can offer a wealth of data on actual market participants who repeatedly perform identical tasks under varying degrees of pressure. For example, several studies examined performance under pressure using penalty kicks in football or shootouts in hockey (Jordet et al., 2007; Kocher et al., 2012; Kolev et al., 2015), while others focus on individual sports such as weightlifting (Genakos and Pagliero, 2012) and tennis (González-Díaz et al., 2012).

In regard to sports, the magnitude of the stakes and the importance of achieving success have been proposed as pressure facilitators (Baumeister, 1984; Kleine et al., 1988). Consequently, critical games in playoffs are a setting where choking expected to evolve.

As choking in various sports has regularly been examined through contestants executing individual sports or closed skills (such as free throws in basketball or penalty kicks in football), and unlike Hill and Shaw (2013) and Hodge and Smith (2014) studies, which used qualitative methods, this study uses a quantitative methodology to provide an exceptional insight into the collective performance.

Therefore, we aim to test if high-profile agents faced with elimination from the playoffs in National Basketball Association (NBA) will exert more effort and overcome choking to outperform their opponents, for whom the game is less critical. The NBA playoffs are a best-of-seven (until 2003 also a best-of-five series were played) elimination tournament that takes place after the end of the regular season among the 16 teams with the highest wins record. The playoffs ultimately converge in single NBA Finals series where the league’s annual champion is determined. In best-of-seven format, a team that accumulates four wins takes the series; this implies that the seventh game, where the score is 3–3, is always critical for both teams. However, games where the score is 3–0 (fourth), 3–1 (fifth), or 3–2 (sixth) are always critical for only one of the teams. One team faced with elimination from the series in the case of loss, whereas the opponent team can afford itself to lose. We hypothesize, based on expected utility theory, that players performing with their back against the wall will exert more effort than their opponents, that is, will win more often.

del Corral et al. (2016) pointed to the high level of competitive balance in the NBA with 17 different teams that reached the NBA finals over the 19 seasons analyzed in the study. The NBA has established policies designed to achieve this objective. This is due to the fact that competitive balance is for long associated with interest from fans and profitability of sports leagues (e.g., Rottenberg, 1956).

In this respect, prolonged playoffs series contribute to the image of competitiveness and generate revenues for the hosting teams from tickets, merchandise and parking; while the NBA organization sells rights to the playoffs broadcasting (Zimmer and Kuethe, 2009; Price et al., 2012). Thus, one may argue that aside from motivation of the team that faced with elimination, the NBA as a whole got a clear financial incentive for a prolonged playoffs series.

## Materials and Methods

The data was collected using standard home computer, from public open source, namely the official NBA website^1^. We scanned through records from playoffs in 33 NBA seasons (1984–2016) and archived data on all games (2542) played during this period. We then sorted out from the sample the 612 games that were played in best-of-five series as they fundamentally different from the best-of-seven configuration and are no longer played in the NBA.

The dataset for analysis comprised from 1930 playoffs games, 437 of these games were critical for either the home or the away team, 77 games were critical for both teams, 1416 games were non-critical for both teams. For this sample the following variables were coded: (1) Type of series [1 = best-of-seven; 0 = best-of-five] (2) Game number in the series [1–7] (3) Home team (4) Critical for the home team [1 = critical; 0 = non-critical] (5) Regular season wins accumulated by the home team (6) Home team won [1 = won; 0 = lost] (7) Away team (8) Critical for the away team [1 = critical; 0 = non-critical] (9) Regular season wins accumulated by the away team (10) Away team won [1 = won; 0 = lost].

We first consider the data in its simplest form, computing the win rates across the seven games of the series for the home teams. Then we present win probabilities conditional on critical/non-critical variable for home and away teams. Finally, we run a binary logistic regression models to predict win probability in critical vs. non-critical games; while accounting for general strength of the teams by entering the number of wins in regular season accumulated by home and away teams.

Balance of power between the teams is a possible confounding factor in our study since weaker teams are more likely to lose games in the series. Consequently, weaker teams will appear more often in games which are critical for them, and they are also more likely to lose those games. Accounting for general strength of the teams in the regression models allows us at least partly to address this concern. On top of that, the ability of the team to generate extra effort in critical situation to overcome even a stronger opponent, for whom the loss is less severe, is exactly the effect we aimed to detect.

## Results

In **Table 1** we present the win probability for the home teams across the seven games of the series.

**Table 1:** Home teams win probability.

	Game number							Total
	1	2	3	4	5	6	7	
Lost	88	87	150	163	71	88	14	661
	25.7%	25.4%	43.7%	47.5%	24.7%	45.4%	18.2%	34.2%
Won	255	256	193	180	216	106	63	1269
	74.3%	74.6%	56.3%	52.5%	75.3%	54.6%	81.8%	65.8%
Total	343	343	343	343	287	194	77	1930
	100.0%	100.0%	100.0%	100.0%	100.0%	100.0%	100.0%	100.0%

The first and the second game in each series are hosted by the team with the better regular season record, that is, team with the home-court advantage in the series. Their opponent then hosts games three and four (until 2003 also game five, i.e., 2-3-2 format). Afterward, game five and game seven hosted by the home-court advantage team (i.e., 2-2-1-1-1 format). The data presented in **Table 1** corresponds with this series configuration, we can see especially high win probabilities for the home teams in games 1, 2, 5, and 7.

As for critical vs. non-critical comparison: the home team won in 945 (66.7%) out of the 1416 games that were non-critical for both teams. The home team won in 137 (74.5%) out of the 184 games that were critical only for the guest team. The home team won in 124 (49.0%) out 253 games that were critical for the home team but not for the guest team, this scenario is the only one where no significant home advantage was recorded.

It may seem that teams tend to underperform in games that are critical to them but not for their opponent; however, we should keep in our mind that teams that face elimination in a series are going to a game with a stronger opponent that outperformed them thus far. In order to account for at least a part of this endogeneity, a binary logistic regression analysis was conducted with the regular season records of the teams entered in the model.

In **Table 2** we controlled for “critical for guest team” variable. The interpretation of the exponential coefficients [the column “Exp(B)”] indicates that each win accumulated during the regular season reflects about 6% higher odds ratio to win for home teams. Similarly, each win accumulated by the opponent team decreases the home teams’ odds ratio to win by around 5%. Playing at home while facing elimination, in games that are non-critical for the guest teams, decreases the odds ratio by around 30%. That means that if the home team got 65% general win probability in playoffs, it will decrease to around 55% in games that are critical for the home team but not for the guest team; such effect exists in the model after the balance of power between the teams reflected by the number of wins in regular season was accounted for.

**Table 2:** Binary logistic regression: home teams’ chances to win in games that are non-critical for the guest teams.

	B	S.E.	Wald	Df	Sig.	Exp(B)
Regular season wins home team	0.057	0.007	62.034	1	0.000	1.058
Regular season wins away team	-0.053	0.007	55.796	1	0.000	0.948
Critical for home team	-0.386	0.145	7.076	1	0.008	0.680
Constant	0.487	0.467	1.088	1	0.297	1.627

We applied the same procedure for the guest team while holding the “critical for home team” variable on zero. Explanatory power of general strength of the teams remained significant (*p* < 0.001) and almost unchanged, whereas, “critical for guest team” variable showed to be negative but non-significant: Exp(B) = 0.759; *p* = 0.132.

**Table 3** presents additional model where predictive power of both the “critical for home team” and “critical for guest team” variables on home teams’ win chances being assessed.

**Table 3:** Binary logistic regression: home teams’ chances to win.

	B	S.E.	Wald	Df	Sig.	Exp(B)
Regular season wins home team	0.058	0.007	74.201	1	0.000	1.060
Regular season wins away team	-0.054	0.007	63.672	1	0.000	0.948
Critical for home team	-0.294	0.131	5.017	1	0.025	0.745
Critical for away team	0.420	0.161	6.818	1	0.009	1.523
Constant	0.435	0.438	0.984	1	0.321	1.545

General strength of the teams remained to be a significant factor in the model presented in **Table 3**, “critical for home team” is negative and significant, analogically “critical for guest team” is positive and significant. The interpretation of the exponential coefficients indicates that playing at home while facing elimination decreases the odds ratio to win by around 25%. When the game is critical for the away team, home teams’ odds ratio to win goes up by around 50%. That means that if the home team got 65% general win probability in playoffs, it will go up to almost 74% in games that critical for the guest team.

The current analysis suggests that team that lost more games in the series thus far and therefore faced with elimination will likely lose another game rather than mobilize itself and outperform its opponent. Two binary logistic regression models presented at **Tables 2**, **3** support this conclusion.

## Discussion

In this paper, we analyzed games in NBA playoffs and demonstrated that the threat of severe losses hinders rather than boosts performance. If boosting effect was taking place we would expect to find that teams facing elimination from the series exert more effort than their opponents that not facing elimination. Yet, our analysis showed that teams playing with their back against the wall lose more often. This negative effect remained significant in the models after we controlled for the general strength of the teams in each playoffs series.

Our current results limit the expected utility theory, which foretell that individuals exert the most effort, and consequently produce their best performances, when the net returns to effort are highest. We demonstrate that above some point the stakes tend to become too high, which corresponds with more than a few recent studies (Dohmen, 2008; Apesteguia and Palacios-Huerta, 2010; Genakos and Pagliero, 2012 as an examples) that used sports to show that when performance matters most individuals feel psychological pressure, and as a result often make uncharacteristic mistakes.

We argue that the setting of critical games in NBA playoffs provided us with an exceptional opportunity to test premises of the expected utility theory in ecologically valid environment where high-profile agents perform under high stakes and with an evident outcome. Initially, we assumed that teams facing elimination would mobilize themselves and try harder than their opponents for which the game is less critical. This, in addition to huge financial incentives for a prolonged playoffs series, led to the hypothesis that one-side elimination games should be won more often by the team playing with the back to the wall.

Instead, our findings are rather in line with Dohmen’s (2008) results on players’ choking during penalty kicks, and demonstrate that the threat of severe losses will not facilitate boosting effect that will lead to elevated level of performance. Yet, Dohmen analyzed single-level performance in a stable environment whereas our results shed light on collective performance in open and dynamic setting. Results from such analysis are relevant to labor economics; thus, we suggest that managers (coaches, or warlords like Xiang Yu) should refrain from deliberate building of high pressure environments with an eye of achieving performance enhancement effect among their groups.

In this regard, Goldman and Rao (2012) elaborate on the asymmetric impact of pressure on performance. Large audiences for instance can produce both negative and positive impact, dependent on the nature of the task. Delicate tasks that require concentration (e.g., free-throw) can be hampered by the same level of pressure that will improve performance of tasks that require power, determination and aggression (e.g., rebounding). Consequently, current results cannot determine at what level the stakes will become too high and the positive correlation between payoff and output will become negative.

Additionally, our findings refute the claims of those who believe that NBA franchises not giving their 100% when they able to eliminate their opponent in one-side elimination game (e.g., Gentelman’s Sweep^2^); or that the NBA organization as a whole manipulating the game in some way in order to generate extra revenues from prolonged series.

## Author Contributions

YG wrote the literature review. EM wrote the methods. YG and EM wrote the results, statistical analysis and conclusions.

## Conflict of Interest Statement

The authors declare that the research was conducted in the absence of any commercial or financial relationships that could be construed as a potential conflict of interest.

## Footnotes

1 https://stats.nba.com/

2 CBSSPORTS.COM: NBA Playoffs preview: Tuesday Night is Gentleman’s Sweep Tuesday https://www.cbssports.com/nba/news/nba-playoffs-preview-tuesday-night-is-gentlemans-sweep-tuesday/
